# *In Situ* Pre-Treatment of Vascularized Composite Allografts With a Targeted Complement Inhibitor Protects Against Brain Death and Ischemia Reperfusion Induced Injuries

**DOI:** 10.3389/fimmu.2021.630581

**Published:** 2021-07-29

**Authors:** Biao Lei, M. Mahdi Sleiman, Qi Cheng, Zhenxiao Tu, Peng Zhu, Martin Goddard, Paulo N. Martins, Logan Langerude, Satish Nadig, Stephen Tomlinson, Carl Atkinson

**Affiliations:** ^1^Division of Hepatobiliary Surgery, The First Affiliated Hospital of Guangxi Medical University, Nanning, China; ^2^Department of Microbiology and Immunology, Medical University of South Carolina, Charleston, SC, United States; ^3^Department of Surgery, Hepatic Surgery Center, Tongji Hospital, Tongji Medical College, Institute of Organ Transplantation, Huazhong University of Science and Technology, Wuhan, China; ^4^Department of Surgery, Hepatic and Vascular Surgery Center, Tongji Hospital, Tongji Medical College, Huazhong University of Science and Technology, Wuhan, China; ^5^Pathology Department, Papworth Hospital NHS Trust, Cambridge, United Kingdom; ^6^UMass Memorial Medical Center, Department of Surgery, Transplant Division, University of Massachusetts, Worcester, MA, United States; ^7^Division of Pulmonary Medicine, University of Florida, Gainesville, FL, United States; ^8^Department of Surgery, Lee Patterson Allen Transplant Immunobiology Laboratory, Medical University of South Carolina, Microbiology and Immunology, Charleston, SC, United States; ^9^Ralph H. Johnson VA Medical Center, Charleston, SC, United States

**Keywords:** vascularized composite allotransplantation, graft treatment, transplantation, complement inhibition, brain death, ischemia reperfusion injury, preservation, immunogenicity

## Abstract

**Introduction:**

Donor brain death (BD) is an unavoidable component of vascularized composite allograft (VCA) transplantation and a key contributor to ischemia-reperfusion injury (IRI). Complement is activated and deposited within solid organ grafts as a consequence of BD and has been shown to exacerbate IRI, although the role of BD and complement in VCA and the role it plays in IRI and VCA rejection has not been studied.

**Methods:**

BD was induced in Balb/c donors, and the VCA perfused prior to graft procurement with UW solution supplemented with or without CR2-Crry, a C3 convertase complement inhibitor that binds at sites of complement activation, such as that induced on the endothelium by induction of BD. Following perfusion, donor VCAs were cold stored for 6 hours before transplantation into C57BL/6 recipients. Donor VCAs from living donors (LD) were also procured and stored. Analyses included CR2-Crry graft binding, complement activation, toxicity, injury/inflammation, graft gene expression and survival.

**Results:**

Compared to LD VCAs, BD donor VCAs had exacerbated IRI and rejected earlier. Following pretransplant *in-situ* perfusion of the donor graft, CR2-Crry bound within the graft and was retained post-transplantation. CR2-Crry treatment significantly reduced complement deposition, inflammation and IRI as compared to vehicle-treated BD donors. Treatment of BD donor VCAs with CR2-Crry led to an injury profile not dissimilar to that seen in recipients of LD VCAs.

**Conclusion:**

Pre-coating a VCA with CR2-Crry in a clinically relevant treatment paradigm provides localized, and therefore minimally immunosuppressive, protection from the complement-mediated effects of BD induced exacerbated IRI.

## Introduction

Vascularized composite allograft (VCA) transplantation (Tx) offers a therapeutic option for treating limb and face loss (1–3). Restoring form and function has the potential to provide major psychological as well as physical benefits (4). However, because of the heterogenicity of tissues, the high immunogenicity of skin, and the procurement of grafts from brain-dead (BD) donors, VCAs generate a strong immunological response post-Tx and require aggressive life-long immunosuppression, which can cause life-threatening side effects (3). In view of this, and because VCA is usually life-changing, but not life-saving, there is an urgent need to develop novel strategies that facilitate improved post-tx outcomes.

While graft rejection is principally dependent on T cells, there are other immune factors that can increase graft antigenicity leading to a strengthening of post-Tx graft injury. Of these, brain death (BD) and ischemia reperfusion injury (IRI) are thought to be the most significant risk factors for subsequent organ dysfunction and rejection. BD-induced injury is associated with increased IRI and alloresponsiveness, and is thought to be a major impediment to tolerance induction. Our knowledge of how these early graft injuries influence post-Tx outcome and the modulation of alloimmunity has been gained largely from studies in solid organ Tx (SOT). The impact of BD on VCA outcomes has not been studied, and little is known regarding how IRI modulates longer-term outcomes after VCA. It has been proposed that composite tissue allografts will be more susceptible to IRI than SOTs because of the heterogenous tissue types (5), although more detailed studies are needed.

Preclinical and clinical models have demonstrated that utilization of grafts from BD donors in SOT is associated with poorer post Tx outcomes (6–15), although the mechanisms involved have not been fully elucidated. BD has been shown to lead to a systemic inflammatory response that promotes donor organ injury and inflammation that pre-sensitizes the graft to increased injury upon reperfusion (16). In recent years, there has been increasing evidence that activation of the complement system plays a key role in the propagation of both BD induced injury/inflammation and IRI (17–19). It is now established that upon reperfusion, the complement system is activated within the graft leading to immune cell infiltration, proinflammatory cytokine production, and graft injury (17, 19–21). We and others have shown that in rodent models, BD leads to complement activation within donor organs during BD, and that complement activation persists even during cold storage (14, 22, 23). Furthermore, these previous studies demonstrated that BD induced complement activation exacerbates post-transplant IRI, and that therapeutic modulation of complement activation in the recipient can improve post-transplant outcomes (15, 20).

The role of complement, BD and how it relates to IRI and rejection in VCA has not been previously investigated. Therefore, in this study, we investigated the effect of donor graft complement inhibition on post-transplant IRI and inflammation in VCAs derived from BD donors. We demonstrate that BD leads to complement activation in the vasculature of the donor VCA, and that BD exacerbates IRI and accelerates the onset of acute rejection. We took advantage of this BD induced donor vascular complement activation and pre-treated BD donor VCAs with CR2-Crry. CR2-Crry is a C3 convertase inhibitor inhibitor that specifically targets to C3 breakdown products that are deposited at sites of complement activation (24). CR2-Crry is comprised of a complement receptor 2 (CR2) targeting domain linked to the complement inhibitor, Crry. CR2 targeting moiety binds to long-lived membrane associated C3 breakdown products, such as iC3b, C3d, and C3dg that are deposited on cell membranes in areas of active complement activation, such as that seen in BD induced complement activation. Crry inhibits all complement pathways at the C3 activation step and inhibits the generation of all biologically active complement activation products (C3 opsonins, C3a, C5a and the membrane attack complex). We perfused BD donor VCA *in-situ* with University of Wisconsin solution augmented with CR2-Crry as a means to pre-coat the VCA prior to graft explant. We demonstrate that CR2-Crry binds to BD donor grafts and is retained within the graft post-Tx. In addition, by pre-coating the graft with CR2-Crry we were able to demonstrate amelioration of BD exacerbated IRI and prolongation of graft survival.

## Materials and Methods

### Animals

Male Balb/c (H-2d) and C57BL/6 (B6; H-2b) mice (8–12 weeks) (Bar Harbor, ME) were used. All procedures were approved by the MUSC Committee for Animal Research (IACUC-01011).

### Brain Death Model

Brain death (BD) induction was performed as described (22). Donor animals with refractory hypotensive episodes, defined as mean arterial pressure <50mmHg for a duration longer than 20 mins, were excluded from the studies, as we have described (22).

### Pre-Transplant Donor VCA Perfusion

Mice were monitored for 3 hrs post BD induction upon which time donor grafts were flushed *in-situ* within the donor and prior to VCA removal, with University of Wisconsin (UW) solution or CR2-Crry (0.13 mg/ml) diluted in 0.5 ml of UW solution. Graft perfusion was performed through the iliac artery for approximately 2 mins, before ligature of the iliac artery and vein, to ensure CR2-Crry was retained within the graft during cold ischemic storage. Grafts were then cold stored for 6 hours at 4°C. For biodistribution studies, animals were randomized into the following 4 groups: 1. Living donor (LD) with UW perfusion, 2. LD with CR2-Crry perfusion, 3. BD donor with UW perfusion, 4. BD donor with CR2-Crry perfusion. For VCA IRI and graft survival studies, the following groups were analyzed 1. LD with UW perfusion, 2. BD donor with UW perfusion, and 3. BD donor with CR2-Crry perfusion. CR2-Crry was produced and purified as described (24).

### Transplant Surgery

The heterotopic hindlimb Tx model was used as described (25), in which Balb/c VCAs were transplanted into fully mismatched C57Bl/6 recipients. For IRI studies, recipient mice were euthanized at 48h post-Tx. For VCA survival studies, mice were monitored daily until rejection was observed, as described (21).

### *Ex Vivo* and *In Vivo* Graft Imaging

CR2-Crry was labeled using XenoLight CF750 NIR (PerkinElmer) according to manufacturer’s instructions. Hindlimb allografts from either LD or BD donors were perfused with CF750NIR-labeled CR2-Crry (or UW as vehicle control) as described above. Maestro II™ *In-Vivo* Fluorescence Imaging System was utilized to detect CR2-Crry in tissues. Two sets of experiments were performed; 1. *Ex vivo* imaging of grafts before Tx at t=0 h and t=6 h after cold static storage and 2. Whole body live animal imaging of recipients after Tx at 6, 24, and 48 h post-Tx. Fluorescence signal quantification was carried out using the Maestro II software by the Small Animal Imaging Unit at MUSC.

### Histopathology and Markers of Injury

Prior to implantation of VCAs, perfusates from cold stored VCAs were collected and levels of creatine kinase (Sigma, USA), and HMGB1 (LifeSpan BioSciences, USA) were measured by ELISA as markers of VCA injury. For histological examination, VCAs were isolated and subsequently fixed, processed to paraffin, and four μm sections stained with Hematoxylin and Eosin (HE) and scored for graft injury from 0-5 using a semiquantitative histology scoring system (26).

### Immunohistochemistry

Graft sections were stained using primary Abs against Nimp-r14 (neutrophils; Thermo Fisher), Mac-3 (macrophages, BD Biosciences), C3d (Complement deposition, R&D), CD3 (T cells, Abcam), CD4 (T helper, Abcam), and CD8 (T cytotoxic, Synaptic Systems). Detection was performed using ImmPRESS™ Kits (Vector Labs) (15). Omission of primary antibody was used as a negative stain control. C3d immunostaining was scored from 0-3 as described (27). Neutrophil and macrophage infiltration was semi-quantitatively assessed using a 3 point scale where 0; few scattered immune cells, 1; intravascular clusters, 2; intravascular and extravascular immune cell clusters, and 3; intravascular and frequent extravascular immune cell clusters associated with areas of tissue damage. All immunohistochemistry scoring was performed by a trained pathologist blinded to the experimental groups.

### RNAScope

mRNA expression was determined using in-situ hybridization (ISH) with the RNAScope 2.5 assay and probes IL-17 and IFNγ (Advanced Cell Diagnostics, Hayward, CA) as described by the manufacturer. Briefly, tissue was pre-treated with heat and protease prior to hybridization of the target probe. Preamplifier, amplifier and an alkaline phosphatase labeled oligos were sequentially hybridized followed by the application of a chromogenic substrate to produce red punctate dots. Tissue was counter-stained with DAPI.

ACD positive control probe (Peptidylprolyl Isomerase B (Cyclophilin B) (PPIB) and negative control probe (B. subtilis gene dihydrodipicolinate reductase) (dapB) were run on each tissue prior to testing with IL-1β, TNFα, IL-17 and IFNγ probes. Tissue passed quality control if the average number of punctate dots per cell throughout the entire sample were greater than five using the PPIB probe and zero using the dapB probe. Fluorescent images were captured using an Keyence BZ-X800 digital slide microscope (Keyence, USA) and ISH scores were generated using intensity of the hybridisation and the distribution throughout the tissue. Images were graded as; 0 (absent), 1 (weak staining in a few areas), 2 (moderate staining in several areas), or 3 (strong staining throughout the tissue).

### Statistical Analysis

GraphPad Prism v7 for Windows (GraphPad, San Diego, CA) was used for statistical analysis. A student t test was used for the comparison of a normally distributed continuous variable between 2 groups. Differences between several groups were compared by the nonparametric Wilcoxon rank-sum or Mann-Whitney test because of the small sample sizes for some experiments. The Mantel Cox text was used to compare survival curves for different groups on univariate analyses. All analyses were 2 sided, and values of P<0.05 were considered statistically significant.

## Results

### Donor Brain Death Induces VCA Complement Deposition

**Figure 1:**
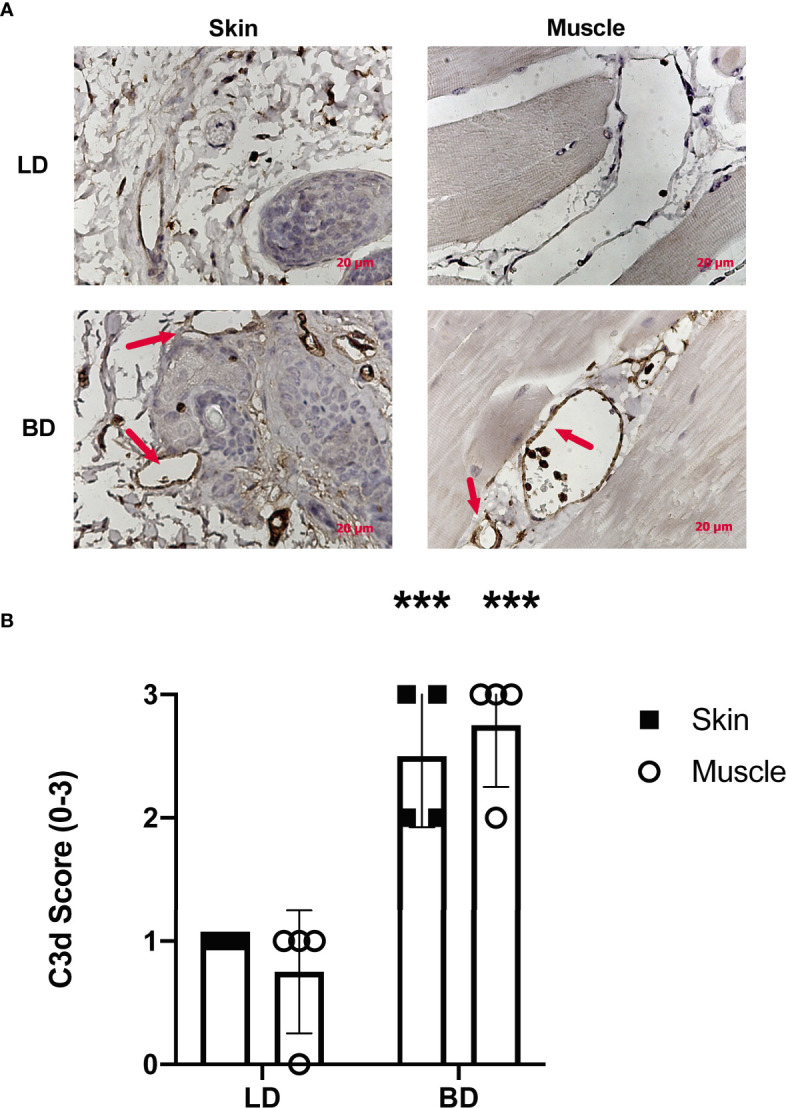
Complement activation is increased in the vasculature of VC donor grafts following brain death. **(A)** Representative images of C3d deposition assessed by immunohistochemistry in living donors and brain dead donors 3 hrs post BD induction. Note the positive staining for C3d (brown) can be seen within vessels of both the skin and muscle in the VC donor graft. Representative images, (n = 4 for each group). **(B)** Semi-quantitative histopathological scoring shows increased C3d deposition in in grafts from brain dead donor tissues as compared to grafts from living donors. Pairwise comparison between brain dead donor *vs* living donor (***p < 0.01, n = 4 in each group).

To assess whether BD lead to activation of the complement system in VCAs, we analyzed skin and muscle tissues in recipient hindlimbs of both LD and BD donors. Complement deposition within the hindlimb was determined by C3d immunohistochemistry. In keeping with previous studies in SOT, 3hrs of donor maintenance post-BD induction led to complement deposition within capillaries, arterioles, and surrounding areas in the muscle and skin. The levels of deposited C3d were significantly higher in BD donors compared to in LD controls (**Figures 1A, B**).

### The C3d-Targeted Complement Inhibitor CR2-Crry Binds and Persists at Higher Levels in Grafts From BD Donors Compared to Grafts From Living Donors

**Figure 2:**
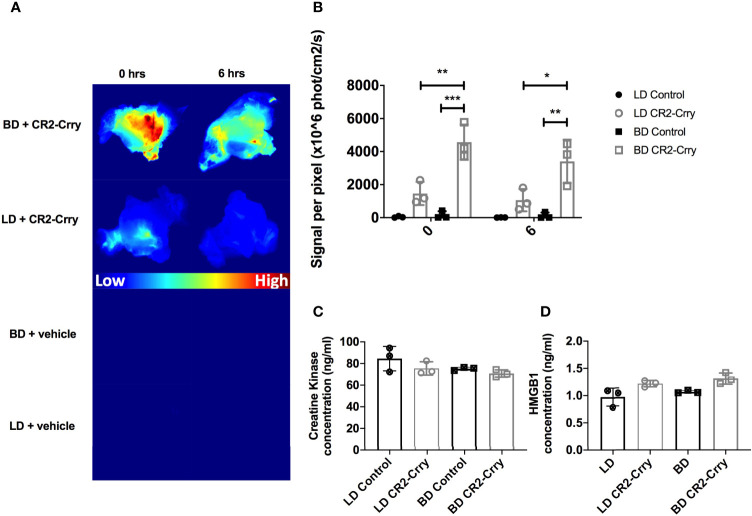
CR2-Crry binds at a higher level in BD donor grafts compared to LD grafts and is not associated with toxicity during cold storage. LD and BD VCAs were perfused with UW or UW augmented with fluorescently labeled CR2-Crry prior to graft harvest and grafts were imaged *ex vivo*. **(A)** Heatmaps showing fluorescence signal intensity of images of grafts at the beginning (t = 0) and end (t = 6hrs) of cold static storage and, **(B)** Their respective signal intensity quantification. Pairwise comparison at t=0 between BD *vs* BD+CR2-Crry (***p < 0.001), LD+CR2-Crry *vs* BD+CR2-Crry (**p < 0.01); difference between LD *vs* LD+CR2-Crry was not significant. Similarly, pairwise comparison at t=6hrs between BD *vs* BD+CR2-Crry (**p < 0.01), LD+CR2-Crry *vs* BD+CR2-Crry (*p < 0.05); difference between LD *vs* LD+CR2-Crry was not significant. Perfusates were collected from VCAs at the end of cold storage were assayed for the concentration of tissue injury markers creatine kinase **(C)** and HMGB1 **(D)** using ELISA. For CK, Pairwise comparisons between LD *vs* BD, LD *vs* LD+CR2-Crry, and BD *vs* BD+CR2-Crry, and LD *vs* BD+CR2-Crry were not significant. The same applies for HMGB1 except for LD *vs* BD+CR2-Crry (*p < 0.05). (n = 3, *p < 0.05, **p < 0.01, ***p < 0.001).

We hypothesized that delivery of CR2-Crry, as a constitute of UW solution, directly to the vasculature at the time of graft harvest would pre-coat the graft and increase its complement inhibitory defense mechanisms. To assess CR2-Crry binding, we perfused grafts *in situ* within the donor with fluorescently labeled CR2-Crry immediately prior to graft harvest and subsequent static cold storage. Imaging of the grafts showed higher fluorescent CR2-Crry binding in grafts from BD donors compared to LDS at both the beginning (t=0) and end (t=6hrs) of cold static storage (**Figures 2A, B**). These data are consistent with immunohistochemical analysis showing increased levels of C3d in grafts from BD donors compared to LD (see above). To determine the impact of BD and static cold storage on graft injury, as well as determine whether CR2-Crry donor therapy had any impact on graft injury during cold storage, we assayed VCA perfusates for high-mobility group box-1 (HMGB1) protein and creatine kinase (CK). These two markers of cell injury and necrosis are well-documented damage-associated molecular patterns (DAMPs) associated with graft and muscle injury, respectively (23, 28). No significant difference was noted in either HMGB1 or CK between any of the groups (**Figures 2C, D**), suggesting that our therapeutic protocol had no impact on early cell viability.

### CR2-Crry Binds and Persists at Higher Levels in Grafts From BD Donors Compared to Grafts From Living Donors Following Transplantation

**Figure 3:**
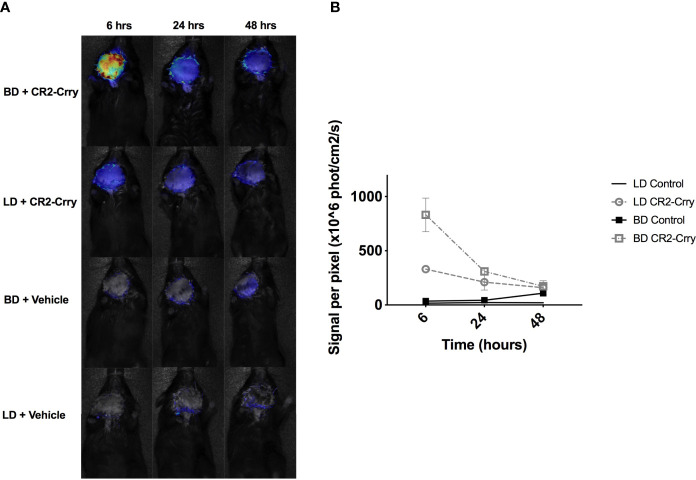
CR2-Crry is retained post-transplantation in VCAs from BD donors. LD and BD VCAs were perfused with UW solution augmented with fluorescently labeled CR2-Crry before VCA procurement. After 6 hrs of cold storage, grafts were heterotopically transplanted into C57BL/6 recipients and fluorescence imaging of whole animals performed at 6, 24, and 48 hrs post-Tx to test for CR2-Crry retention in grafts. **(A)** Recipient mouse photos overlaid with heatmaps of CR2-Crry fluorescence intensity at different timepoints and **(B)** Respective quantification of signal intensity. Multiple t-test, one per timepoint, were performed between LD+CR2-Crry *vs* BD+CR2-Crry. CR2-Crry signal is significantly elevated at 6 hours post transplantation in BD *vs* LD CR2-Crry treated grafts. (n = 3 for CR2-Crry treated groups, n = 1 for controls).

We next determined whether CR2-Crry remained bound within the graft following transplantation. LD or BD donor grafts were perfused as described above with either UW alone or UW augmented with fluorescently labeled-CR2-Crry. For clinical relevance, grafts from all groups were cold static stored for 6 hrs a 4°C prior to heterotopic transplantation in C57BL/6 fully mismatched recipients. Using live animal imaging, recipients were imaged at 6, 24, and 48 hrs post-transplantation to quantitatively assess CR2-Crry graft retention. BD donor grafts showed higher levels and persistence of CR2-Crry at 6 hours post-Tx compared to LD grafts. CR2-Crry signal decreased by 24 hrs and was almost undetectable by 48 hours after transplantation (**Figures 3A, B**).

### Perfusion With CR2-Crry Protects Grafts From Brain Death Exacerbated Ischemia Reperfusion Injury

**Figure 4:**
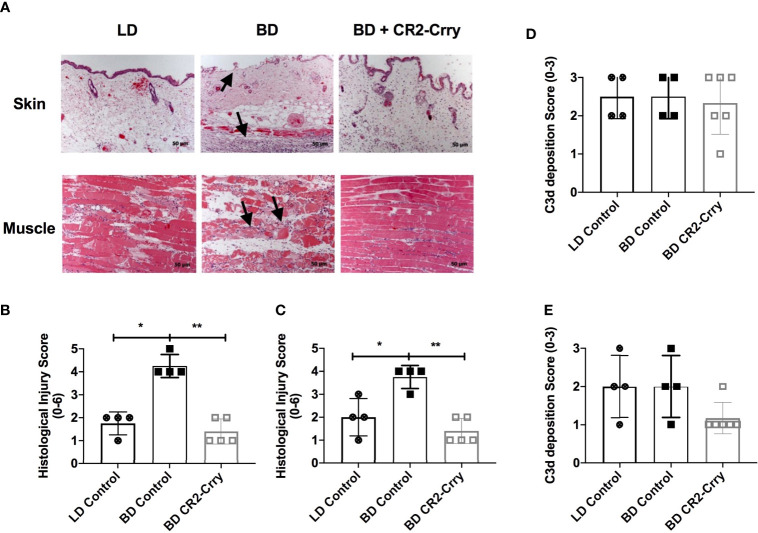
Donor graft treatment with CR2-Crry reduces histological evidence of graft injury in VCAs at 48 hours post-Tx. **(A)** Histological sections of skin and muscle graft tissue collected 48 hours post transplantation and stained with H&E. Sections were assessed for injury using a semi-quantitative histopathological score by blinded observer. Note the immune infiltrates in the muscle and skin, and associated muscle and epithelial injury seen in BD (arrows) as compared to LD and BD+CR2-Crry. Pairwise comparison for both skin **(B)** and muscle **(C)** between LD *vs* BD (*p < 0.01), and BD *vs* BD+CR2-Crry (**p < 0.01); differences between LD *vs* BD+CR2-Crry were not significantly different. Quantification of C3d deposition measured by immunohistochemistry staining of skin **(D)** and muscle **(E)** VCA graft tissues sections. No significant difference in C3d deposition could be determined at 48 hrs post transplantation.

VCAs explanted 48 hrs post-transplant from recipients that received either LD or BD donor grafts exhibited key features associated with IRI, including muscle and epidermal damage, inflammatory cell infiltration, and endothelial activation. However, histological scores of injury and inflammation were higher in both muscle and skin compartments from recipients that received VCAs from BD donors compared to VCAs from LDs (**Figures 4A, B**). Complement is known to play a role in BD and IRI of SOT, and since the above data show that IRI is exacerbated in VCAs from BD donors, we investigated how UW solution supplemented with CR2-Crry, and administered at the time of VCA donor procurement, impacted IRI in BD donor VCAs.

We opted to focus these therapeutic studies on BD donors only given that: 1. All VCAs are derived from BD donors clinically and, 2. CR2-Crry requires C3d deposition in the donor graft for binding and optimum function. BD donors treated with CR2-Crry supplemented UW solution prior to graft harvest and after 6 hrs of cold storage exhibited reduced histopathological evidence of graft injury at 48 hrs post-transplantation as compared to untreated BD controls (**Figures 4A–C**). Of note, CR2-Crry BD donor treatment reduced both muscle and skin graft injury to levels at or below that seen in LD VCA controls (**Figures 4A–C**).

In a previous report using a rodent model of cardiac transplantation, we demonstrated that BD exacerbates complement deposition post transplantation (15). We therefore investigated whether the exacerbated injury seen in BD donor VCAs after transplantation is associated with increased C3d deposition. Immunohistochemistry demonstrated the presence of C3d in all grafts analyzed, with staining present on endothelial cells lining capillaries, muscle, and epithelial cells (not shown). Quantification of C3d deposition revealed no significant difference in C3d deposition in either the skin or muscle of VCAs when comparing LD *vs* BD, and BD *vs* BD + CR2-Crry (**Figures 4D, E**).

**Figure 5:**
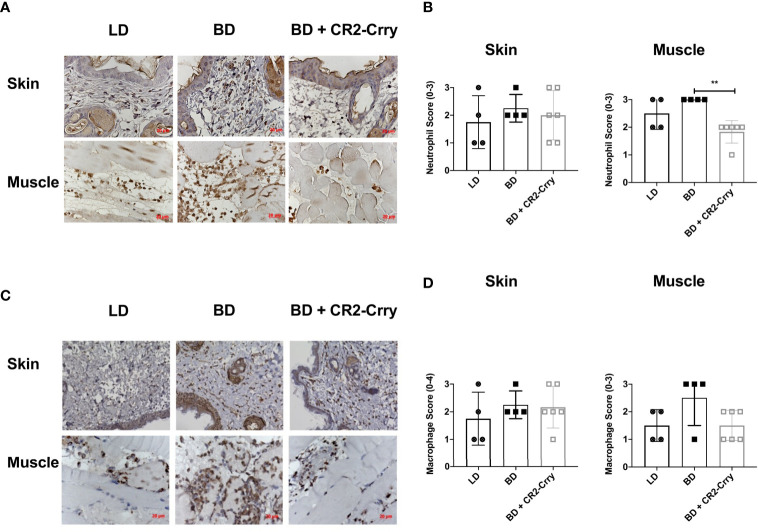
Pretransplant perfusion of BD donor grafts with CR2-Crry reduces immune cell infiltration at 48 hours post-transplantation. **(A)** Representative images of muscle and skin sections stained by IHC for neutrophils (Nimp). **(B)** Quantification of neutrophil infiltration in skin and muscle, showing a reduction in neutrophils in muscle of BD CR2-Crry treated donors as compared to BD control group (**p = 0.008). **(C)** Representative images of muscle and skin sections stained by IHC for macrophages (Mac-3). **(D)** Quantification of macrophage infiltration in skin and muscle, showing no significant reduction in macrophages in both skin and muscle of BD CR2-Crry treated donors as compared to BD or LD control groups.

Complement activation plays a key role in endothelial cell activation and chemotaxis of innate and adaptive immune cells. To determine whether BD donor complement therapy reduced innate immune cell infiltration, we analyzed neutrophil and macrophage infiltration using immunohistochemistry. All grafts had neutrophil and macrophage infiltrates as determined by Nimp-r14 (neutrophil) and Mac-3 (macrophages) immunoreactivity. Semi-quantitative histopathological scoring revealed that neutrophil infiltration was not significantly different between any of the tested groups in the skin (**Figures 5A, B**). Analysis of muscle tissues from VCAs demonstrated a significant reduction in neutrophil infiltration in BD VCAs treated with CR2-Crry as compared to untreated BD and LD grafts (**Figures 5A, B**). A similar reduction was seen with macrophage scoring with CR2-Crry treatment of BD grafts, however no significant differences was observed between any of the groups (**Figures 5C, D**).

To determine whether BD donor complement therapy reduced adaptive immune cell infiltration, we analyzed the infiltration of CD3, 4 and 8 T cells using immunohistochemistry. While CD3, 4 and 8’s where present in muscle and skin, they were sparsely distributed and no significant differences was noted between any of the groups.

### CR2-Crry Significantly Reduced IL-17 Intragraft Gene Expression

**Figure 6:**
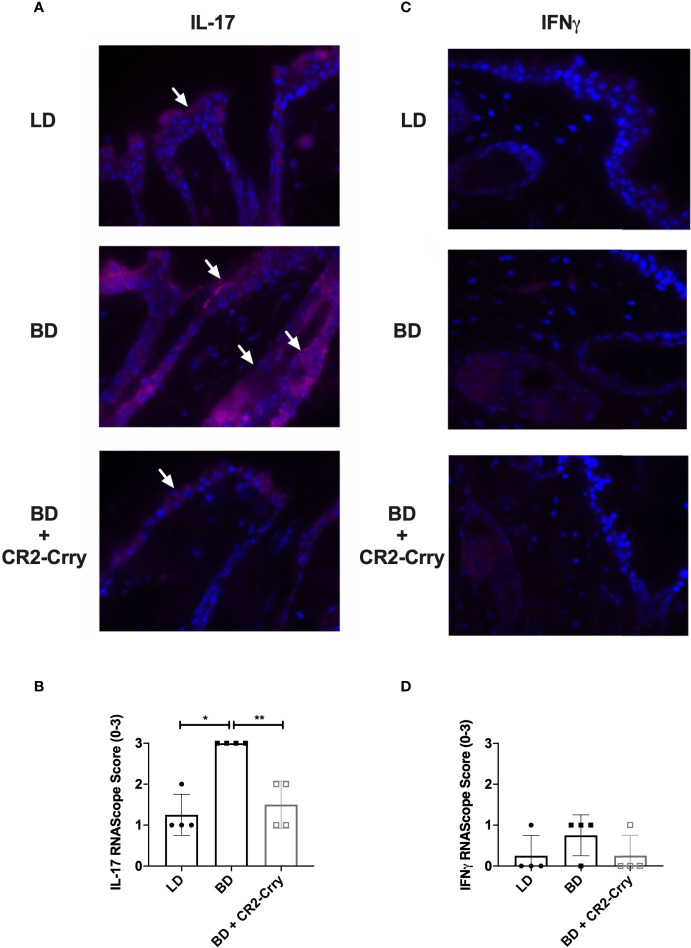
CR2-Crry donor therapy reduces intragraft IL-17 mRNA expression. Representative images from n = 4/group of IL-17 and IFNγ RNAScope *in-situ* hybridization **(A, C)**. Positive mRNA signal (red) for IL-17 was seen in the squamous epithelim of all groups **(A)**, but was quantitatively increased in recipients of brain dead donor grafts **(B)**. No significant mRNA for IFNγ was noted in the epithelium or immune cells in any group **(C, D)**. Images x60 magnification, n = 4 all groups. Pairwise comparison for IL-17 between LD *vs* BD (*p < 0.03), and BD *vs* BD+CR2-Crry (**p < 0.04); differences between LD *vs* BD+CR2-Crry were not significantly different.

Intragraft cytokine expression was analyzed using RNAScope assay in skin samples from all groups. Analysis of IL-1β and TNFα gene expression was not significantly different between groups (data not shown). Complement activation has been shown to drive IL-17 and IFNγ expression by immune cells and epithelial cells, expression of which has been associated with graft injury and rejection (29). IFNγ mRNA expression was seen in macrophages and immune cells in both the skin (**Figure 6**), although significant differences in expression were not seen. IL-17 mRNA was seen in squamous epithelium of skin and in immune cells scattered through out the epidermis. Semi-quantitative analysis of mRNA levels showed the recipients of BD donor grafts had significantly more IL-17 expression than LD controls. Further, IL-17 mRNA levels were significantly reduced in recipients of CR2-Crry pre-treated BD donor grafts as compared to BD controls (**Figure 6**).

### Donor Brain Death Significantly Reduces Allograft Survival

**Figure 7:**
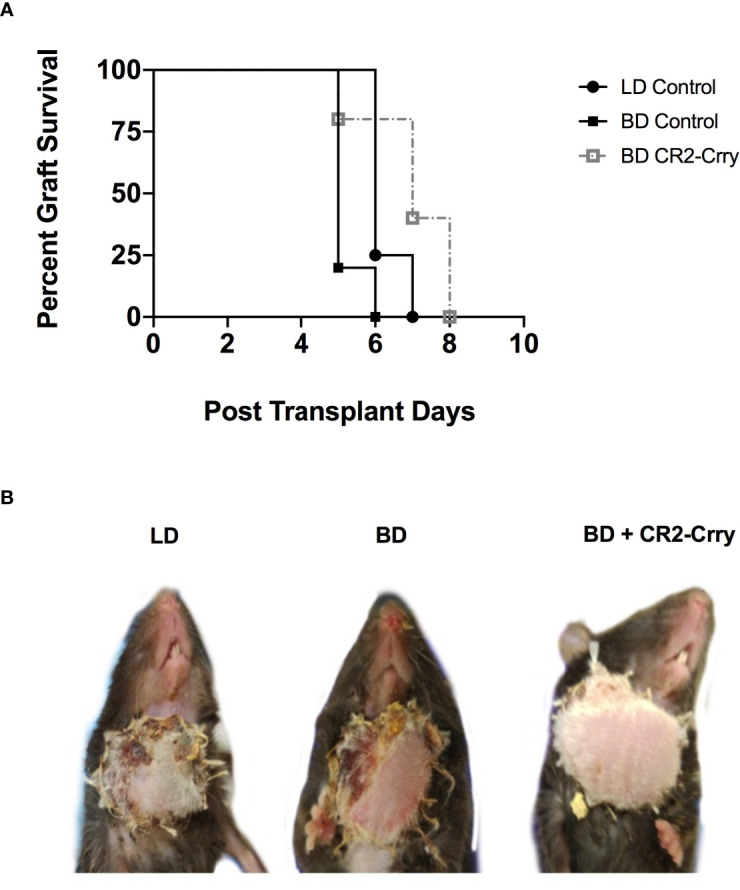
Impact of BD and CR2-Crry BD donor pre-treatment on VCA survival. **(A)** VCA graft survival curve. **(B)** Representative images of recipient mice at day 5 post-Tx. Logrank tests comparing survival across the 4 groups demonstrated survival times were significantly different between groups (p < 0.0001). All pairwise comparisons demonstrated that survival was significantly greater among each group when compared to the BD group (for LD *vs* BD, and for BD *vs* BD+CR2-Crry), even after a Holm-Sidak adjustment for multiple comparisons. No significant difference was seen between living BD or BD+CR2-Crry. (n = 4-6).

To further assess the impact of BD on outcomes after VCA transplantation, as well as to assess the therapeutic potential of BD donor delivered targeted complement inhibition in a clinically relevant setting, we performed VCA survival studies. VCAs from LD and BD BALB/c donors were transplanted into C57BL/6 recipients. In concordance with our IRI data above, BD was associated with significantly poorer median graft survival as compared to grafts from a LD (**Figures 7A, B**). Treatment of BD donor grafts with CR2-Crry augmented UW solution significantly improved graft survival as compared to grafts from untreated BD and LD controls (**Figures 6A, B**). Logrank test suggested that survival times were significantly different among groups (p<0.0001). All pairwise comparisons demonstrated that survival was significantly greater among each group when compared to the BD group (p<0.01 for all), even after a Holm-Sidak adjustment for multiple comparisons. No significant difference was seen between living or BD + CR2-Crry donor groups.

## Discussion

While a number of studies have been conducted in solid organ transplantation (30–34), to date no studies have investigated brain death experimentally in the context of vascularized composite allografts. Here we demonstrate for the first time that the vasculature of vascularized composite allografts from brain dead donors have a significantly higher level of deposited complement activation products compared to vascularized composite allografts from living donor controls. We further demonstrate that following transplantation, vascularized composite allografts from brain dead donors have increased ischemia reperfusion injury and shorter graft survival.

Inhibitors that systemically inhibit complement, such as sCR1 and anti-C5 antibody, have been investigated in experimental models and in the clinic (35–38). These studies in solid organ transplantation demonstrate that complement inhibition is anti-inflammatory and protects against acute post-transplant injury. However, these strategies require systemic delivery to the recipient and systemic complement inhibition, and the earliest they can be administered is at the time of reperfusion. While protective, these approaches likely provide suboptimal protection given that they would need time to systemically neutralize complement. Of further importance, an additional level of immunosuppression with systemic complement inhibition is of special infection concern for vascularized composite allografts recipients that will be heavily immunosuppressed. We have shown that targeting complement inhibitors to the graft by systemic administration of complement receptor 2 (CR2)-targeted complement inhibitors, CR2-Crry and CR2-fH, inhibits IRI with minimal effect on systemic complement levels (21). Nevertheless, all these complement inhibitory strategies require systemic delivery and cannot be administered until the time of reperfusion; this likely provides suboptimal protection compared to a strategy that allows treatment at the time of graft procurement with retention of graft protectant in the recipient (39, 40).

Pratt et al. (19), and Yu et al. (40), using rat kidney transplant models, investigated a unique approach to target complement inhibitors to the donor kidney *ex-vivo*, by including it as a constituent of the organ preservation solution. Pre-treatment of grafts with a lipid-tagged CR1, a C3 inhibitor, resulted in a reduction in early damage and prolongation of graft survival (19). Delivery of an anti-C5 monoclonal antibody or TT30, a humanized CR2 targeted-factor H construct that specifically inhibits the alternative pathway of complement, both improved graft function and survival, although anti-C5 monoclonal antibody performed better than TT30. It was not clear from the presented data whether TT30 bound within the kidney. Given that the study used kidneys from living donors, it is unclear whether C3 ligands for CR2 were present at the time of TT30 infusion into the donor kidney. The authors did clearly show an increase in C3 ligands as a consequence of cold static storage, and that this was reduced in TT30 treated kidneys, it was not clear whether this was associated with TT30 inhibition in the fluid phase or at a bound surface (40). Taken together, these studies highlight the potential of pre-coating or pre-treating the donor graft with a complement inhibitor as a means to bolster the complement inhibitory defenses of the graft.

Vascularized composite allografts present a unique opportunity in donor management given that the perfusion of these grafts can be isolated in the BD donor without impacting other organs. Here we show that donor brain death activates complement in a vascularized composite allograft, and therefore took the innovative approach to utilize C3 ligands deposited in the graft as a consequence of brain death, and that are directly involved in driving injury, to target CR2-Crry to the vasculature of vascularized composite allografts in the brain dead donor prior to graft removal. Following delivery of CR2-Crry as a constituent of UW solution, we demonstrated that CR2-Crry was retained within the graft during cold static ischemic storage, and that binding was significantly higher in vascularized composite allografts from brain dead donors compared vascularized composite allografts from living donors. These data, together with data showing high levels of C3d deposited in graft from brain dead donors but not living donors, also demonstrate that C3 ligand availability is essential for CR2-Crry retention. Importantly, we flushed the living donor and brain dead donor vascularized composite allografts at the end of 6 hrs of cold storage period so that only graft-bound CR2-Crry would be detected. CR2-Crry was also retained at high levels within the graft at 6 hrs post transplant, but was largely lost by 24 hrs post-transplant.

Brain death exacerbated IRI is mediated, among other factors, by the infiltration of innate immune cells that enter the graft through inflammatory cues such as complement anaphylatoxins and cytokine/chemokine signals. Using an ordinal scoring system, when compared against untreated vascularized composite allografts from brain dead donors, we observed trends for a reduction in neutrophil and macrophage infiltration, and complement deposition in vascularized composite allografts from living donors and in vascularized composite allografts from brain dead donors treated with CR2-Crry. However, the only significant difference noted was a decrease in neurophil infiltration in the muscle compartment of vascularized composite allografts from CR2-Crry treated brain dead donors. In our previous VCA study, CR2-Crry was administered to the recipient at 0 and 24 hrs post-transplantation, and significant reductions in neutrophil and macrophage infiltration were observed (21). We had anticipated finding a similar response in the current study. On the other hand, our *in-vivo* imaging data showed that CR2-Crry was largely lost from the grafts by 24 hrs post-transplant, and thus while analysis of cell infiltration at 48 hrs post transplantation enabled us to compare outcomes with the published literature, particularly how brain death impacts ischemia reperfusion injury, it may have been too late to determine associations of brain dead donor therapy with immune infiltrates and graft complement deposition, which temporally may start to peak after loss of complement control. Supplementary treatment of the recipient with CR2-Crry may provide an additional level of protection, but the goal of this study was to determine whether donor therapy alone provided graft protection.

The extent of graft injury early post-transplantation mediated by brain death-induced and ischemia reperfusion injury are thought to promotes adaptive immune cell priming and rejection (34, 41, 42). Here we show that brain death increases the tempo of acute rejection of vascularized composite allografts, and further that pre-treatment of the brain dead donor grafts prior to cold storage with graft-targeted CR2-Crry significantly prolongs graft survival. In addition to injury signals and inflammatory cytokines, complement activation has been shown to play a direct role in promoting alloimmunity. C3a and C5a anaphylatoxins can skew T cell responses and promote the generation of alloreactive T cells (43–45). To this end we investigated T cell infiltration in grafts at 48 hrs. We did not see any significant differences in numbers of T cells between any of the groups, and in general T cells infiltrates were sparse.

Acute rejection in vascularized composite allografts has been shown to be characterized by an IFNγ and IL-17 mediated response (29). Therefore, here we determined intragraft IFNγ and IL-17 mRNA expression at 48 hrs post transplantation. No significant IFNγ expression was noted, but there was a significant upregulation of IL-17A. IL-17 (IL-17A), a signature cytokine produced by a subset of T helper cells and plays essential roles in host defense against bacterial and fungal infections. However chronic overproduction of IL-17 contributes to inflammatory skin conditions like psoriasis (46). While IL-17 is predominantly produced by immune cells, IL-17A mRNA expression has been described in inflamed epithelial cells of the skin, colon and lung (47–49). Here we show significant increases in IL-17A mRNA in epithelial cells in brain dead donors as compared to all other groups. IL-17 plays important roles epithelial barrier functions but it also drives inflammation and priming of immune cells that likely contribute to graft rejection. C3a and C5a have been previously shown to induce upregulation of IL-17 secretion by epithelial cells in lung transplantation (47). Therefore, increased complement activation seen in brain dead donors may drive IL-17 over production and priming of alloimmunity. Taken together further studies are needed to investigate the interplay of complement with intragraft cytokine expression.

In summary, pre-coating a vascularized composite allograft with CR2-Crry in a clinically relevant treatment paradigm provides localized, and therefore minimally immunosuppressive, protection from the complement-mediated effects of brain death induced exacerbation of ischemia reperfusion and acute rejection.

## Data Availability Statement

The original contributions presented in the study are included in the article/supplementary material. Further inquiries can be directed to the corresponding authors.

## Ethics Statement

The animal study was reviewed and approved by MUSC IACUC Committee.

## Author Contributions

BL, MS, QC, ZT, LL and PZ performed experimental procedures, data interpretation. MG, PM, and SN data interpretatation and manuscript preparation. Conceptual design, data interpretation, and manuscript preparation: CA and ST. All authors contributed to the article and approved the submitted version.

## Funding

These studies were supported by grants from the NIH (NIAID 1U01 AI132894-01, 1R56AI156383-01 to CA/ST), the Department of Defense (RW81XWH-16-1-0783 to CA, W81XWH2010743 to ST), American Heart Association (18PRE34070023 to MS), and 111 Projects (D17011 to BL).

## Conflict of Interest

ST is an inventor on a licensed patent for CR2-targeted complement inhibition.

The remaining authors declare that the research was conducted in the absence of any commercial or financial relationships that could be construed as a potential conflict of interest.

## Publisher’s Note

All claims expressed in this article are solely those of the authors and do not necessarily represent those of their affiliated organizations, or those of the publisher, the editors and the reviewers. Any product that may be evaluated in this article, or claim that may be made by its manufacturer, is not guaranteed or endorsed by the publisher.

## Abbreviations

BD, brain death; CR2, complement receptor 2; IR, ischemia reperfusion; IRI, ischemia reperfusion injury; LD, living donor; Tx, transplantation; VC, vascularized composite; VCA, vascularized composite allotransplantation; HMGB1, high-mobility group box-1; CR2-Crry, Complement Receptor 2-Crry; SOT, Solid organ transplant.
